# The First Report on Strategic Management of Bilateral Carotid Artery Stenosis: Exploring Surgical Challenges and Decision‐Making

**DOI:** 10.1002/ccr3.70803

**Published:** 2025-08-18

**Authors:** Meghdad Ghasemi Gorji, Yasamin Bigdeli, Kimia Jazi

**Affiliations:** ^1^ Shiraz University of Medical Sciences Shiraz Iran; ^2^ Student Research Committee, Faculty of Medicine Qom University of Medical Sciences Qom Iran; ^3^ School of Medicine, Shahid Beheshti University of Medical Sciences Tehran Iran

**Keywords:** bilateral carotid artery stenosis, bilateral stenosis management, carotid artery disease, carotid endarterectomy (CEA)

## Abstract

Bilateral carotid artery stenosis is a significant risk factor for ischemic stroke, with a prevalence of 8%–39% among stroke patients. This report highlights a challenging case that highlights the complexities of treating bilateral carotid artery stenosis. A 62‐year‐old male with a history of significant stenosis in the right carotid artery, previously treated with stenting and angioplasty, presented with a transient ischemic attack exhibiting right side symptoms, indicating stenosis on the left side. CT angiography revealed 99% stenosis in the right common and internal carotid arteries and 75% stenosis in the left carotid artery. Given the patient's previous history of stenting, carotid endarterectomy was selected as the optimal treatment choice. Despite challenges including the extensive length of stenosis and the presence of a previous stent, the procedure was successfully completed with restored blood flow. This case emphasizes the importance of individualized surgical planning that considers anatomical and clinical factors in managing bilateral carotid artery stenosis. The decision‐making process highlights how tailored approaches can lead to optimal outcomes in complex vascular cases.

**Summary**

- This case emphasizes the complexity of managing bilateral carotid artery stenosis, highlighting the importance of a tailored surgical approach. The decision to address the asymptomatic right stenosis first, due to the risk of ischemia from the severe left stenosis, underscores the need for careful, individualized treatment planning in high‐risk patients.

## 1 Introduction

Bilateral carotid artery stenosis is a well‐known significant risk factor for ischemic stroke, with a prevalence among stroke patients ranging from 8% to 39% [1]. Treatment options include carotid endarterectomy (CEA) and carotid artery stenting (CAS) performed unilaterally or bilaterally [1]. Managing this condition remains a debate. Both interventions entail a significant risk of periprocedural stroke, hemodynamic instability, and cerebral hyperperfusion syndrome [2]. The Carotid Revascularization Endarterectomy vs. Stenting Trial (CREST) compared the efficacy of CAS and CEA. The results showed no considerable difference between the two treatments; however, the study highlighted varied periprocedural risks, such as a higher incidence of stroke associated with CAS, while CEA had a higher incidence of myocardial infarction [3]. Moreover, a randomized controlled trial demonstrated that although both procedures showed the same efficacy, CAS had a higher periprocedural risk of stroke or death compared to CEA in patients with symptomatic carotid stenosis, particularly above 70 years old [4].

A systematic review and meta‐analysis conclude that bilateral CAS could be a safe strategy with lower costs and reduced treatment time in cases presenting with bilateral stenosis [2]. Nevertheless, there is no consensus or a guideline for the timing, procedure technique, and strategy (staged vs. simultaneous) regarding patients' situations in bilateral stenosis. Accordingly, the challenge remains whether the right or left artery should be stented first. Herein, we report a 62‐year‐old man with a history of significant right carotid artery stenosis, stent placement, and angioplasty, who presented with right side motor weakness, proposing another cerebrovascular accident (CVA) on the left brain. This discrepancy between the location of the stenosis and the side affected by the stroke provided valuable insight into the patient's vascular condition and placed us against a great challenge in the surgical approach.

## 2 Case History

**FIGURE 1:**
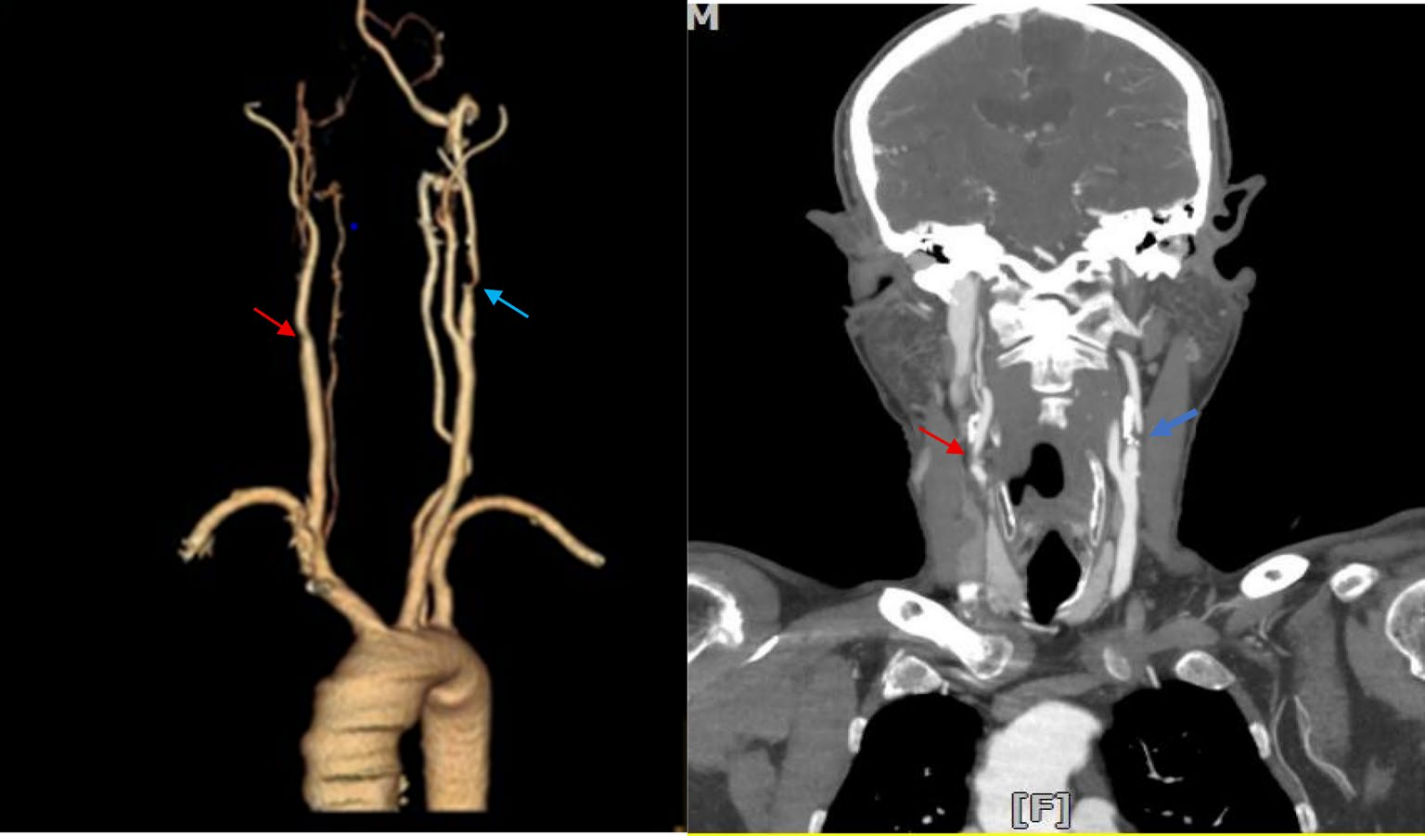
Preoperative CT angiography (CTA) image showing 99% stenosis in the right common and internal carotid arteries (indicated by red arrows) and the previously implanted stent in this area. Additionally, 75% stenosis is observed in the left carotid artery (blue arrow), which was associated with neurological symptoms (motor weakness in the right side of the body).

A 62‐year‐old male with a history of transient ischemic attack (TIA) and CVA 2 years ago, for which he underwent CAS on the right side, was presented to our clinic complaining of novel transient symptoms. He had been consistently taking his prescribed medications, including aspirin 80 mg daily, clopidogrel 75 mg daily, and statins 40 mg daily. The patient complained of sudden severe motor weakness in the right upper extremity and mild weakness in the right lower extremity, which lasted for approximately 30 min and occurred 2 weeks prior to his current visit. These symptoms were consistent with a new TIA that had occurred 2 weeks ago. A CT angiography (CTA) performed at an outside facility revealed 99% stenosis in the right common carotid artery and internal carotid artery, as well as 75% stenosis in the left carotid artery (Figure 1). Given the severity of bilateral carotid artery disease, a precise and careful treatment strategy was required.

## 3 Methods

The challenge in this case was that if the symptomatic left‐sided stenosis were treated first with temporary carotid artery occlusion during surgery, there would be a high risk of cerebral ischemia due to the near‐complete (99%) stenosis on the right side. This critical blockage in the right carotid artery severely limited collateral circulation, and the patient's brain was highly dependent on the remaining blood flow from the left carotid artery. The electroencephalogram monitoring (EEG) was employed during the procedure to continuously assess cerebral function and detect any signs of cerebral ischemia. To ensure adequate cerebral perfusion during the cross‐clamping of the carotid artery, an intraoperative shunt was inserted, and EEG monitoring was continued to monitor neurological status throughout the procedure. Stump pressure monitoring was not utilized in this case. Instead, backflow from the internal carotid artery was evaluated as an indicator of collateral circulation, and saline washing was performed prior to arterial clamping and shunt placement to minimize the risk of embolic events.

## 4 Conclusion and Results

**FIGURE 2:**
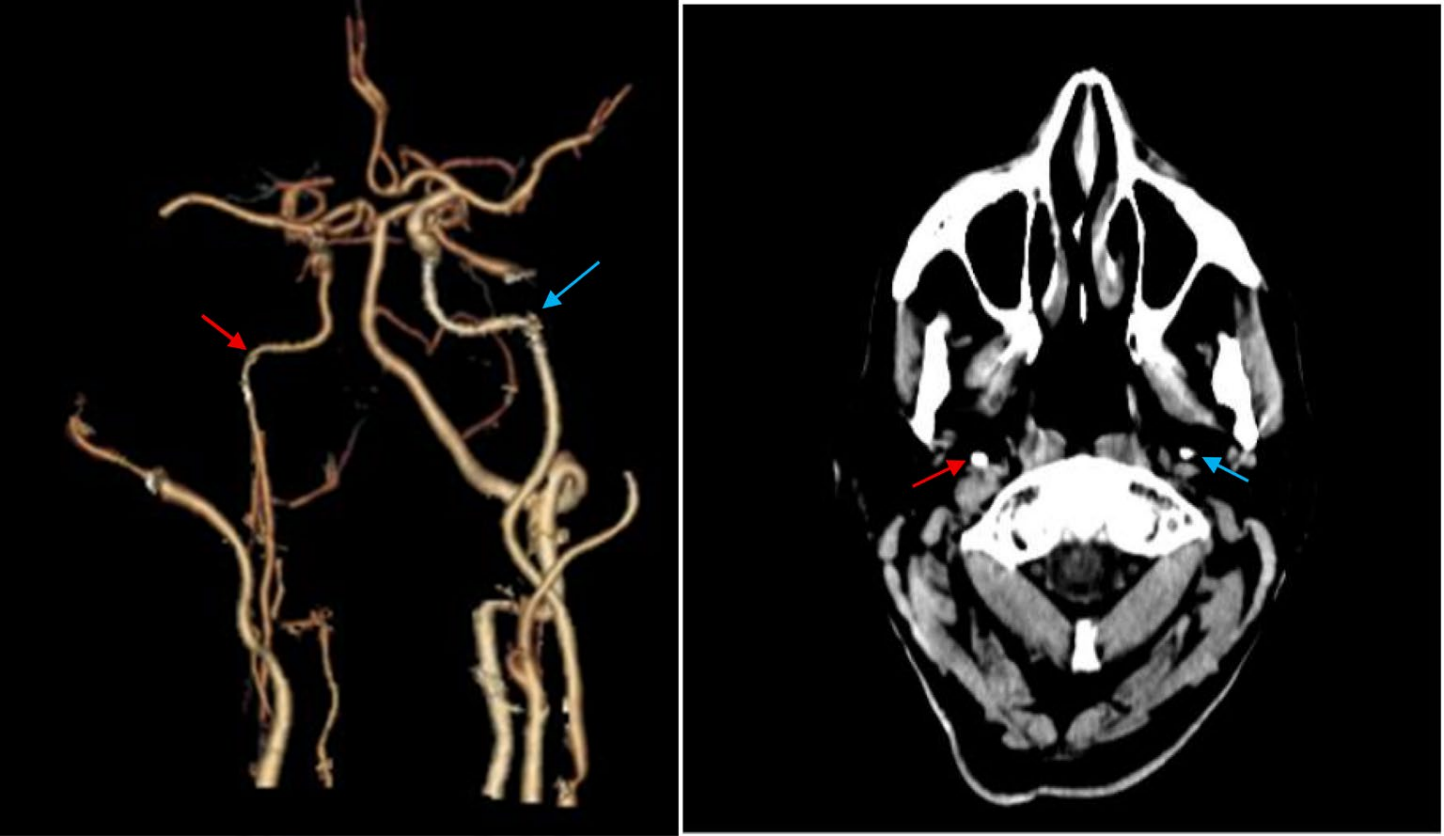
Postoperative CTA image of the right carotid artery demonstrating successful stent removal and complete vascular reconstruction (red arrow). No residual stenosis or complications are observed. Postoperative CTA image of the left carotid artery showing persistent 75% stenosis (blue arrow), which is scheduled for intervention in the second stage.

Thus, surgery was initially performed on the right carotid artery, which had 99% stenosis but was asymptomatic (Figure 2). The patient was categorized as having moderate cardiovascular risk based on standard preoperative assessments. A thorough cardiac evaluation prior to surgery revealed no significant dysfunction. Additionally, the patient maintained stable and consistent cardiac performance in the postoperative period without any signs of deterioration.

This surgery presented complexities due to the extensive length of the stenosis, which made access and treatment challenging, as well as the presence of a prior stent, which required extra care to avoid damage to the vessel or displacement of the stent.

Despite these challenges, the surgical team opted to perform CEA on the right side. After controlling the vessels, the common carotid artery and internal carotid artery were carefully examined. A longitudinal arteriotomy was performed, and the extensive plaque was successfully removed. To repair the artery and restore normal blood flow, a long patch from the patient's saphenous vein was sewn into the common carotid artery, which helped improve blood flow and prevent further stenosis at the repair site. Postoperatively, adequate blood flow through the right carotid artery has been established. The patient was hospitalized for 5 days, and after receiving the necessary treatments, was discharged on therapeutic‐dose heparin, apixaban 15 mg twice daily, and pantoprazole 40 mg, in addition to the previously prescribed medications.

Following the successful treatment of the right‐sided stenosis, surgery for the left carotid artery stenosis was scheduled for 2 months later; allowing sufficient time for recovery and ensuring adequate blood flow to the brain from the newly opened right carotid artery.

## 5 Discussion

Stroke is one of the leading causes of death worldwide, affecting nearly 13.7 million people annually. About 80% of strokes are caused by reduced blood flow in the brain, leading to significant ischemia [5]. Carotid artery stenosis accounts for approximately 12% of ischemic strokes [1]. Bilateral carotid stenosis refers to the presence of 60% or greater diameter narrowing in the internal and/or common carotid arteries on both sides, or the presence of 60% or greater diameter narrowing in the left internal and/or common carotid artery accompanied by 60% or greater narrowing in the innominate artery [6]. Bilateral carotid stenosis is a complex condition, and the optimal treatment strategy for it remains a subject of debate. There are limited approaches available for treating these patients, including:

- Performing carotid surgery (CEA) either in a staged manner or simultaneously.
- Angioplasty or stenting in the carotid artery (CAS)
- Or a combination.

The best treatment approach should be determined based on the individual patient's condition. Factors to consider include the patient's symptoms, the type of plaque in the artery, the structure of the aortic arch, the anatomy of the internal carotid artery (ICA), and comorbidities [7, 8, 9, 10, 11, 12, 13].

Rapid advancements in endovascular techniques and the introduction of distal embolic prevention devices and stent designs have made CAS a suitable option for patients with bilateral stenosis. This method carries a lower incidence of cranial nerve palsy and myocardial infarction (MI) compared to simultaneous bilateral CEA, which poses a risk of severe complications due to injury to the phrenic, pharyngeal, and vagus nerves. In other words, CAS is a lower‐risk approach for treating bilateral carotid stenosis [8].

Most vascular surgeons prefer to perform CEA in a staged manner due to concerns about the negative hemodynamic effects from stimulation of the carotid sinus after stenosis correction, as well as the risk of cerebral hyperperfusion syndrome. Noteworthy, surgeons aim to reduce the potential risks associated with this treatment method [14]. CEA is recommended for most symptomatic patients with internal carotid artery stenosis between 50% and 99%, as well as for patients with asymptomatic stenosis between 60% and 99%. This procedure has a death rate and perioperative stroke rate of < 3%. On the other hand, in cases where symptomatic patients have internal carotid artery stenosis between 50% and 99%, there is restenosis after CEA, and there are medical or anatomical contraindications for surgery; CAS should be considered. In other words, CEA is a suitable option for treating carotid artery stenosis, while CAS is recommended in other specific circumstances [15]. Recent literature supported both CEA and CAS for patients with symptomatic or asymptomatic carotid stenosis [16]. According to the guidelines of the American Academy of Neurology and the Asymptomatic Carotid Atherosclerosis Study (ACAS), endarterectomy is recommended for patients with asymptomatic carotid stenosis, provided they are between 40 and 75 years old and are expected to live at least 5 years after the surgical procedure [17, 18].

The CREST study has shown that patients who undergo endarterectomy are at a higher risk of myocardial infarction. This increased risk is due to the greater stress placed on the heart (myocardium) during the surgery. In other words, the pressure and strain exerted on the heart during the surgical procedure can increase the likelihood of cardiovascular complications, particularly above their 70s [3]. This increased risk is due to the complexity and increased tortuosity of the access vessels (the vessels accessed during the surgical procedure). In other words, at older ages, the anatomical features of the vessels can contribute to a higher likelihood of stroke occurrence [18]. The recommended criteria for performing stenting based on the results of the NASCET and SAPPHIRE studies include the following: restenosis after endarterectomy, occlusion of the contralateral internal carotid artery, presence of severe cardiopulmonary disease, and patients at high surgical risk [19].

CEA and CAS have their advantages and disadvantages, and only careful selection of the patients with important preoperative planning can tailor the choice. However, challenges still exist in patient selection and the best timing for revascularization. Treatment of bilateral carotid disease with nearly 8%–39% prevalence among patients with symptomatic carotid stenosis is one of the most undiscussed questions. Bilateral carotid stenosis is still a relative contraindication to CEA and is excluded from most prospective trials [20].

In this case, the patient presented with symptomatic 75% stenosis at the distal common carotid artery and the left internal carotid artery, as well as recurrent asymptomatic carotid stenosis that had previously undergone stenting and angioplasty (99% stenosis in the distal common carotid artery and the right internal carotid artery). The decision was made to perform an endarterectomy on the right carotid artery to prevent cerebral ischemia on the right side. We performed the endovascular intervention only on the right carotid artery, as performing a bilateral procedure in one surgery is contraindicated due to the high risk of mortality and perioperative or postoperative stroke. More importantly, the almost occluded right carotid artery could threaten the patient's survival if we clamp the left carotid first for placing a left side stent according to reduced blood flow and insufficient collaterals. This procedure was successful, and the symptoms related to carotid stenosis decreased. The left carotid artery stent is planned for next month. The particularity of our case is that 99% stenosis was observed in the right common and internal carotid arteries, as well as 75% stenosis in the left side (which had already resulted in a stroke). If we had treated the symptomatic stenosis on the left side first and temporarily clamped the carotid artery, given the 99% stenosis on the opposite side, cerebral ischemia would almost certainly have occurred, reducing the patient's survival. Therefore, we first operated on the right side, which had 99% stenosis but was asymptomatic. This surgery was challenging due to the extensive length of the stenosis and the presence of a previous stent. Taking on the risk, we controlled the artery, explored the common and internal carotid arteries as much as possible, performed a longitudinal incision to remove the stenosis, and completed the endarterectomy. We then placed a long saphenous vein patch on the common carotid artery and restored blood flow. The surgery on the left side is scheduled for next month.

In this case, given the patient's prior history with CAS, CEA was deemed the most appropriate and safe option for management. The extensive stenosis and presence of a previous stent necessitated meticulous surgical planning to avoid vessel damage or stent displacement. To mitigate the risk of cerebral ischemia during cross‐clamping, intraoperative EEG monitoring was employed to continuously assess cerebral function, and a shunt was placed to ensure adequate cerebral perfusion. Notably, stump pressure monitoring was not utilized; instead, collateral circulation was evaluated through backflow assessment from the internal carotid artery, and saline washing was performed prior to clamping and shunt insertion to minimize embolic risks. These measures were critical in addressing the unique challenges posed by the patient's bilateral stenosis.

Preoperative cardiac evaluation categorized the patient as moderate cardiovascular risk, with preserved cardiac function. Postoperatively, the patient maintained stable cardiac performance without deterioration, underscoring the importance of thorough preoperative assessment and individualized perioperative management in high‐risk cases.

## 6 Conclusion

Currently, we reported a symptomatic left side CVA patient presented with bilateral carotid stenosis; the right side was almost occluded and 75% of the left. Deciding on the surgical approach and which side to address first placed us against challenges. Treatment selection should be based on the severity of the stenosis, clinical symptoms, carotid anatomy, associated comorbidities, and the overall condition of the patient. Surgical methods and interventional treatments, alongside medical management, can yield favorable outcomes in improving the patient's prognosis.

## Author Contributions

**Meghdad Ghasemi Gorji:** conceptualization, investigation, supervision, writing – review and editing. **Yasamin Bigdeli:** data curation, investigation, methodology, resources, writing – original draft, writing – review and editing. **Kimia Jazi:** investigation, writing – review and editing.

## Ethics Statement

This case report was conducted in accordance with the principles of the Declaration of Helsinki. All efforts were made to maintain the patient's privacy and confidentiality.

## Consent

Written informed consent was obtained from the patient for the publication of this case report, including clinical details and images.

## Conflicts of Interest

The authors declare no conflicts of interest.

## Data Availability

No datasets were generated or analyzed during the current study. However, the patient's further clinical evaluations during this study are available from the corresponding author upon reasonable request.
